# Effect of Roasting and Brewing on the Antioxidant and Antiproliferative Activities of Tartary Buckwheat

**DOI:** 10.3390/foods9091331

**Published:** 2020-09-21

**Authors:** Ji-yeon Ryu, Yoonseong Choi, Kun-Hwa Hong, Yong Suk Chung, Somi Kim Cho

**Affiliations:** 1 School of Biomaterials Sciences and Technology, College of Applied Life Sciences, SARI, Jeju National University, Jeju 63243, Korea; rjo211@naver.com; 2 Lucy Cavendish College, University of Cambridge, Cambridge CB3 0BU, UK; yc472@cam.ac.uk; 3 With O Co., Jeju 63309, Korea; witho62@naver.com; 4 Department of Plant Resources and Environment, Jeju National University, Jeju 63243, Korea; yschung@jejunu.ac.kr; 5 Interdisciplinary Graduate Program in Advanced Convergence Technology and Science, Jeju National University, Jeju 63243, Korea

**Keywords:** antioxidant, antiproliferative, brewing, roasting, Tartary buckwheat

## Abstract

We evaluated the effect of the roasting and brewing conditions of Tartary buckwheat (TB), which is widely used in infusion teas, on its antioxidant and antiproliferative activities in vitro. TB was roasted at 210 °C for 10 min and brewed at a high temperature for a short time (HTST; 85–90 °C, 3 min) or at room temperature for a long time (RTLT; 25–30 °C, 24 h). Roasted TB (RTB) tea brewed at RTLT had the highest total polyphenol content (TPC) and total flavonoid content (TFC) among the four TB teas for different roasting and brewing conditions. Moreover, RTB brewed at RTLT showed the greatest 2,2-diphenyl-1-picrylhydrazyl-, 2,2’-azino-bis(3-ethylbenzothiazoline-6-sulphonic acid)-, and alkyl-scavenging activities. The TB tea brewed at RTLT had higher Fe^2+^-chelating activity than that brewed at HTST, irrespective of roasting. Moreover, RTB tea brewed at RTLT inhibited the proliferation of human pancreatic and breast cancer cells. Overall, RTB-RTLT displayed the largest effect on antioxidant and antiproliferative effects. Finally, rutin was found to possess the most pronounced effect on the antioxidant and antiproliferative activities of the TB teas. These results indicate that the antioxidant and antiproliferative activities of RTB are enhanced by RTLT brewing.

## 1. Introduction

Buckwheat is a type of crop that belongs to the *Fagopyrum* genus and Polygonaceae family. There are two major species of buckwheat with agricultural importance: common buckwheat (*F. esculentum* Moench.) and Tartary buckwheat (TB; *F. tataricum* Gaertn.). Buckwheat cultivation originated in the area of central Tibet and Northern Pakistan. Recognized for its health benefits, buckwheat is widely cultivated throughout temperate Europe, Russia, China, and the Himalayas [1]. Buckwheat is an important source of bioactive compounds that exert pharmacologically demonstrated antioxidant, antiproliferative, antihypertensive, and antidiabetic effects [2]. Buckwheat is rich in flavonoids (e.g., rutin, orientin, quercetin, vitexin, isovitexin, and isoorientin) and vitamins (vitamin B1, B2, and E) [1,3]. Among these compounds, rutin is regarded as being responsible for the pharmacological activities of buckwheat because buckwheat contains a higher level of rutin (3–8%) than any other medicinal plant [4]. Moreover, TB contains greater quantities of these useful compounds, particularly rutin, than does common buckwheat [3,4]. Thus, TB has recently garnered much attention as a dietary source of antioxidants [4,5].

Reactive oxygen species (ROS) are a byproduct of normal cellular metabolism and exposure to various exogenous sources of oxidants (e.g., cigarette smoke, ozone, and ionizing radiation) [6]. The physiologically important ROS include O^2−^, H_2_O_2_, and OH radicals [7]. When present in excess, these ROS can directly or indirectly damage proteins, lipids, carbohydrates, and DNA [8]; this condition is termed oxidative stress. Oxidative stress, a state in which the oxidant/antioxidant balance has shifted in favor of oxidants, is related to several pathological conditions such as cancer, neurological disorders, atherosclerosis, hypertension, diabetes, and aging [9]. These harmful effects of ROS are counteracted by antioxidants [7], including phytochemicals. Phenolic compounds, a class of bioactive phytochemicals, scavenge intracellular ROS, thereby reversing their pathological effects [10].

Brewing is an extraction process that depends on a number of variables, including the volume and temperature of the water, brewing time, and the material grind size [11]. Brewing affects not only the phytochemical composition of tea but also its antioxidant activity [12,13,14]. The effects of cold brewing on the phenolic compounds and antioxidant activities in coffee and tea have been investigated [11,15,16]. However, most studies of brewing methods have focused on coffee and green tea; a few studies have investigated the effect of cold processing methods on the antioxidant activity of TB. Hence, we investigated the effect of hot and cold brewing (otherwise known as Dutch extraction), with or without roasting, on the antioxidant and antiproliferative activities of TB, and we identified and quantified the active components.

## 2. Materials and Methods

### 2.1. Chemicals and Reagents

The following phenolic compounds were purchased from Sigma-Aldrich (St. Louis, MO, USA). Folin–Ciocalteu phenol reagent, 2,2-diphenyl-1-picrylhydrazyl (DPPH), 2,2’-azino-bis (3 ethylbenzothiazoline-6-sulphonic acid) (ABTS), 2,2’-azobis(2-methylpropionamide) dihydrochloride (AAPH), and α-(4-pyridyl-1-oxide)-*N*-tert-butylnitrone (4-POBN) were purchased from Sigma Chemical Co. (St. Louis, MO, USA). Dulbecco’s modified Eagle’s medium (DMEM), trypsin/ethylenediaminetetraacetic acid (EDTA), fetal bovine serum (FBS), and 100× penicillin/streptomycin solution were purchased from Gibco (Grand Island, NY, USA). Dimethyl sulfoxide (DMSO) and 3-(4,5-dimethylthiazol-2-yl)-2,5-diphenyltetrazolium bromide (MTT) were purchased from Amresco (Solon, OH, USA). Solvents for high-performance liquid chromatography (HPLC) were purchased from Merck, Inc. (Darmstadt, Germany). All chemicals and reagents were of analytical grade.

### 2.2. Roasting of Buckwheat

TB (*F. tataricum* (L.) Gaertn.) was provided by Prof. Yong Suk Chung, Jeju National University, South Korea in September 2018. Buckwheat (1 kg) was roasted at 210 °C for 10 min to obtain a medium roast using a coffee roaster (PROASTER, Taehwan Automation Ind, Co., Bucheon, Korea). Non-roasted TB (NRTB) and TB roasted at 210 °C (RTB) were used. Husked TB was then pulverized in a household milling machine for 30 s to yield fine particles of buckwheat powder.

### 2.3. Extraction

#### 2.3.1. Cold Brewing

Cold brewing was carried out at room temperature (20–25 °C). A sample of 150.0 g of TB was placed in 1 L of mineral water in a jar with a screw-top lid. Water (at room temperature) was slowly dripped onto a panel at a rate of one drop per 10 s, and the TB was allowed to brew for 24 h.

#### 2.3.2. Hot Brewing

**Figure 1:**
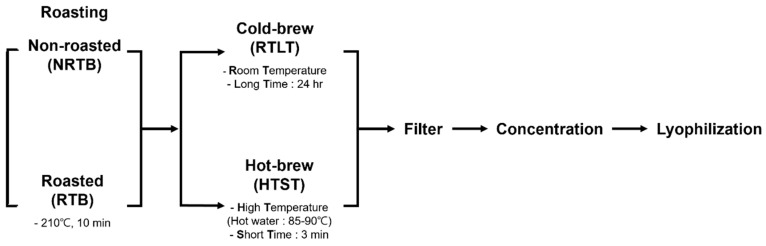
Schematic of the preparation of Tartary buckwheat (TB) teas.

Hot brewing was performed as described by Saklar, with slight modification [17]. Hot brewing was conducted using the same buckwheat-to-water ratio as for cold brewing (Figure 1). A sample of TB was placed in a teabag, and boiling (85–90 °C) water was then added. This experiment was designed to mimic a typical brewing environment. Thus, filtering was conducted for 3 min. Next, the samples were passed through filter paper and concentrated in a vacuum rotary evaporator under reduced pressure at 40 °C. Finally, the samples were frozen at −80 °C, lyophilized, and stored at 4 °C until required. Extraction yields were determined in wt %. To minimize the effect of beverage quality, all samples were prepared using the same brand of commercial mineral water.

### 2.4. Determination of Total Polyphenol and Flavonoid Contents

The total polyphenol content (TPC) was assessed as described by Cheung et al. (2003), with slight modifications [18]. An aliquot (125 μL) of the extract was mixed with 0.5 mL of the Folin–Ciocalteu phenol reagent and incubated for 5 min. Following 5 min of incubation, 1 mL of 10% (*w/v*) Na_2_CO_3_ was added to the reaction mixture and allowed to proceed for 30 min in the dark, after which the absorbance at 700 nm was measured using a microplate reader (Sunrise; Tecan, Salzburg, Austria). Results are expressed as milligrams of gallic acid equivalent (GAE) per gram of dry sample. The total flavonoid content (TFC) was measured using the method described by Zhishen et al., (1999) with slight modification [19]. An aliquot (40 μL) of the extract was mixed with 80 μL distilled water and 6 μL of 5% NaNO_2_. Following 5 min of incubation, 12 μL of 10% AlCl_3_ was added and incubated for 6 min. A volume of 40 μL of 1 N NaOH and distilled water was then added to the mixture and the absorbance was recorded at 510 nm using a microplate reader. Results are expressed as milligrams of rutin equivalent (RE) per gram of dry sample.

### 2.5. Assessment of Antioxidant Activities

#### 2.5.1. Fe^2+^-Chelating Activity Assay

Fe^2+^ chelating activity was assessed according to Chung et al. (2003), with minor modifications [18]. Briefly, 100 μL of the extract were added to 20 μL of 2 mM FeCl_2_, 40 μL of 5 mM ferrozine, and 640 μL of ethanol and for 10 min at room temperature. Finally, absorbance at 562 nm was measured using a microplate reader. Chelating activity was quantified as the percentage reduction in absorbance using EDTA (50 and 100 μM) as the positive control.

#### 2.5.2. DPPH-Radical-Scavenging Activity Assay

DPPH-radical-scavenging activity was evaluated as previously described [20,21]. DPPH radical solution (160 μL of 200 μM), and 40 μL of the extract was dispensed into a 96-well plate (catalogue S0096, SPL Life Sciences, Pocheon, Korea) and incubated 37 °C for 30 min. Following incubation, absorbance was measured using a microplate reader at 517 nm. Scavenging activity was quantified as the percentage reduction in absorbance using catechin (100 and 200 μM) as the positive control.

#### 2.5.3. ABTS-Radical-Scavenging Activity Assay

ABTS-radical-scavenging activity was determined as described previously [22]. ABTS radical cation solution (7 mM ABTS in 2.45 mM potassium persulfate) was prepared and incubated at room temperature for 20 h. The stock solution was then diluted with distilled water to an absorbance of 0.700 ± 0.005 at 734 nm and measured using a UV1800 spectrophotometer (Shimadzu, Kyoto, Japan). An aliquot (100 μL) of the extract was mixed with 900 μL of diluted stock solution and incubated for 2 min. Scavenging activity was quantified as the percentage reduction in absorbance using α-tocopherol (100 and 200 μM) as the positive control.

#### 2.5.4. Alkyl-Radical-Scavenging Activity Assay

Alkyl radical solution was prepared by mixing 40 mM AAPH and 40 mM 4-POBN [23]. Alkyl radical solution and the extract were then incubated at 37 °C in a water bath for 30 min and transferred to 50-μL Teflon™ capillary tubes. Alkyl-radical-scavenging activity was assessed using a JES-FA200 electron spin resonance (ESR) spectrometer (JEOL, Tokyo, Japan) provided by the Bio-Health Materials Core-Facility in Jeju National University (Jeju, South Korea) with the following parameters: magnetic field, 336.00 mT; power, 7 mW; sweep time, 30 s; sweep width, 10 mT; frequency, 9.43 GHz; modulation width, 0.2 mT; and time constant, 0.03 s. The signal intensity was compared to that of the magnetic ESR standard (Mn^2+^ marker) and is presented as the relative height ratio. Catechin (10 and 20 μM) was used as the positive control.

### 2.6. Assays of Antiproliferative Activity

#### 2.6.1. Cell Culture

Human pancreatic (MIA PaCa-2, Panc-1) and breast (MCF-7, MDA-MB-231) cancer cells were obtained from the Korean Cell Line Bank (Seoul, South Korea). The cells were cultured in DMEM containing 10% heat-inactivated FBS and 1% antibiotics and were maintained in a humidified incubator at 37 °C in a 5% CO_2_ atmosphere.

#### 2.6.2. Cell Viability Assay

Cell viability was determined by the MTT assay [24]. Briefly, cells (2 × 10^4^/mL) were seeded in 96-well culture plates and incubated for 24 h. Following incubation for 24 h, the cells were treated with 0, 250, 500, and 1000 μg/mL of the extracts. Next, 20 μL of MTT solution (5 mg/mL) was added to each well, and the supernatant was removed and 150 μL of DMSO was added to each well and the absorbance measured at 570 nm using a microplate reader. Cell viability was quantified as the percentage reduction in absorbance.

### 2.7. Quantification of Phenolic Compounds

An ultrafast liquid chromatograph with a diode array detector (UFLC-DAD; Shimadzu) equipped with a shim-pack GIS column (250 mm × 4.6 mm, 5-μm ODS; Shimadzu, Kyoto, Japan), a quaternary pump, and an autosampler was used for quantifications. Formic acid of 0.1% (*v/v*) in distilled water (A) and acetonitrile (B) at a flow rate of 0.8 mL/min was used as the mobile phase. Starting with 100% of solvent A, an isocratic elution was run for 5 min followed by a gradient elution set as follows: 5–10 min, 3% B; 10–15 min, 5% B; 15–20 min, 10% B; 20–25 min, 20% B; 25–30 min, 30% B; 30–35 min, 30% B; 35–40 min, 20% B; 40–45 min, 10% B; 45–50 min, 0% B; and 50–55 min, 0% B. The injection volume was 40 μL and the oven temperature was set to 30 °C. The detector was set to 254 nm.

### 2.8. Statistical Analysis

All experiments in the present study were performed in triplicate, and means and standard deviations were calculated using the SPSS software (version 18.0; SPSS, Inc., Chicago, IL, USA). Differences among groups were evaluated by the least significant difference (LSD) of a one-way analysis of variance. Pearson’s product-moment correlation was used to obtain relationships of phytochemical contents with antioxidant and antiproliferative activities. A biplot was drawn using Stat Graphics software (StatPoint Technologies, Warrenton, VA, USA).

## 3. Results and Discussion

### 3.1. Extraction Yields, Total Polyphenol, and Flavonoid Contents of TB Teas

The extraction yields, TFC, and TPC of the TB teas are shown in Table 1. The yields of RTB (room temperature for a long time (RTLT) and high temperature for a short time (HTST), 4.81% and 0.28%, respectively) were higher than those of NRTB (RTLT and HTST, 2.12% and 0.23%, respectively). Similarly, Elsorady and Ali (2018), reported that the yield of roasted peanut skin was higher than that of unroasted peanut skin [25]. In this study, the extraction yield of RTLT was 9.22- and 17.18-fold higher than that of HTST. This differs from a previous report indicating that cold-water extraction of steaming green tea resulted in a lower yield than did hot water extraction [15]. Thus, the extraction yield could be influenced by factors other than time, such as temperature.

Phenolic compounds, which include phenolic acids, flavonoids, and coumarins have potent antioxidant activity [26]. Therefore, we determined the TPC and TFC of the TB teas. RTB brewed at RTLT exhibited the highest TPC and TFC values (11.07 ± 0.55 mg GAE/g dry weight and 6.03 ± 0.93 mg RE/g dry weight, respectively), followed by RTB brewed at HTST (4.98 ± 0.44 mg GAE/g dry weight and 3.47 ± 0.84 mg RE/g dry weight), NRTB brewed at HTST (4.83 ± 0.40 mg GAE/g dry weight and 2.59 ± 0.51 mg RE/g dry weight), and NRTB brewed at RTLT (3.58 ± 0.31 mg GAE/g dry weight and 1.86 ± 0.28 mg RE/g dry weight). Notably, RTB brewed at RTLT had higher and NRTB brewed at RTLT had lower TFC and TPC compared with the other TB teas. These results suggest that roasting influences the TPC and TFC of TB brewed at RTLT, but not that brewed at HTST.

### 3.2. Antioxidant Activities of TB Teas

**Figure 2:**
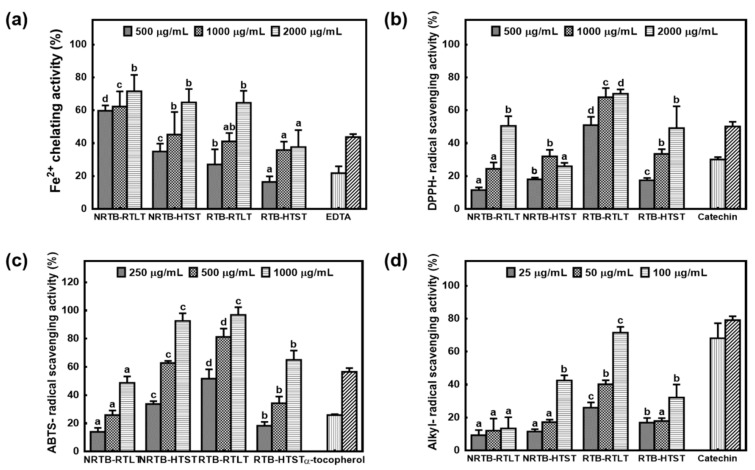
Antioxidant activities of TB teas. (**a**) Fe^2+^-chelating activities, (**b**) DPPH-radical-scavenging activities, (**c**) ABTS-radical-scavenging activities, and (**d**) alkyl-radical-scavenging activities. The appropriate quantities of EDTA, catechin, and α-tocopherol were used as the positive controls. NRTB, non-roasted Tartary buckwheat; RTB, roasted Tartary buckwheat. RTLT, room temperature (25–30 °C) for 24 h; HTST, high temperature (85–90 °C) for 3 min. Values are mean ± SD (*n* = 3). Different letters represent significant differences among extracts, as determined by the least significant difference test at *p* < 0.05. DPPH, 2,2-diphenyl-1-picrylhydrazyl; ABTS, 2,2’-azino-bis (3 ethylbenzothiazoline-6-sulphonic acid); EDTA, ethylenediaminetetraacetic acid.

ROS play an important role in the development of diseases such as cancer, aging, and cardiovascular conditions. Antioxidants scavenge ROS to prevent and reverse the damage caused by oxidative stress. The Fe^2+^-chelating activity of NRTB brewed at RTLT (59.74–71.54% at 500–2000 μg/mL) was higher than that of the other TB teas (Figure 2a). Interestingly, however, the antioxidant activities of the TB teas were inconsistent with their TPC and TFC values. Similarly, Chinnapun (2018) reported that raw samples of bambara groundnut seeds had higher metal-chelating activities than heated samples [27]. The DPPH-scavenging activities of RTB brewed at RTLT were 51.00–70.09% at sample concentrations of 500–2000 μg/mL, followed by NRTB brewed at RTLT (11.52–50.52%), RTB brewed at HTST (17.46–49.15%), and NRTB brewed at HTST (18.03–32.00%; Figure 2b). The ABTS-radical scavenging activities of TB teas increased in a sample-concentration-dependent manner (Figure 2c). The RTB brewed at RTLT showed the greatest radical-scavenging activities (51.65–96.86%), followed by NRTB brewed at HTST (33.64–92.56%), RTB brewed at HTST (18.28–61.94%), and NRTB brewed at RTLT (13.95–48.67%). In addition, ESR spectrometry was performed to measure the alkyl-radical scavenging activities of TB teas. For this, we generated alkyl-radicals at 37 °C for 30 min using AAPH and 4-POBN and analyzed the spin adducts by ESR spectrometry. The alkyl-radical-scavenging activity of RTB brewed at RTLT was significantly higher than that of the other TB teas (26.00–71.49% at 25–100 μg/mL; Figure 2d). Rao and Fuller (2018) reported that cold-brew coffee has lower antioxidant activity than hot-brew coffee [16]. However, RTB brewed at RTLT had markedly greater alkyl-radical-scavenging activity than the other TB teas. This may be due to the effects of brewing parameters other than water temperature on the extraction of bioactive compounds.

### 3.3. Antiproliferative Activities of TB Teas

**Figure 3:**
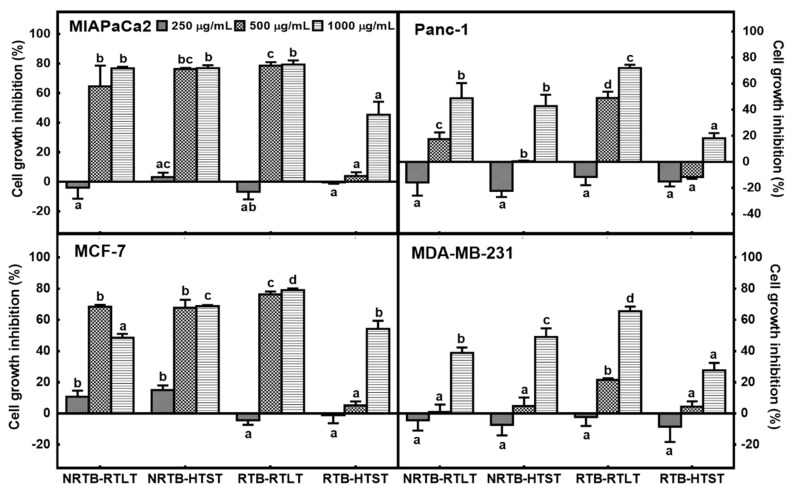
Antiproliferative activities of TB teas. Effect of TB teas on the proliferation of human pancreatic cancer (MIAPaCa2 and Panc-1) and breast cancer (MCF-7 and MDA-MB-231) cells as measured by an MTT assay. NRTB, non-roasted Tartary buckwheat; RTB, roasted Tartary buckwheat. RTLT, room temperature (25–30 °C) for 24 h; HTST, high temperature (85–90 °C) for 3 min. Values are mean ± SD (*n* = 3). Different letters represent significant differences among extracts, as determined by the least significant difference test at *p* < 0.05. MTT, 3-(4,5-dimethylthiazol-2-yl)-2,5-diphenyltetrazolium bromide.

Plants rich in antioxidant phenolic compounds are beneficial for cancer prevention, and the anticancer activity of TB phytochemicals has been evaluated in human breast, hepatoma, and lung cancer cells [28,29]. Therefore, we investigated the effects of the brew method and roasting condition of TB teas on the proliferation of human pancreatic and breast cancer cell lines. The magnitude of the inhibition by RTB brewed at RTLT of the proliferation of MIAPaCa2, Panc-1, MCF-7, and MDA-MB-231 cells was greater than that of the other TB teas (Figure 3). In particular, the growth of Panc-1 and MDA-MB-231 cells was significantly inhibited in a dose-dependent manner by RTB brewed at RTLT (*p* < 0.05). Notably, the antiproliferative activity of HTST differed markedly according to the roasting conditions. The antiproliferative activity of tea is closely related to its phenolic contents. Li et al. (2017) reported that the antiproliferative activity of phenolics in TB is influenced by their combination with other phytochemicals [29]. Thus, we evaluated the phenolic contents of the TB teas.

### 3.4. Phenolic Contents of the TB Teas

The concentrations of six phenolic compounds (three phenolic acids, two flavonols, and flavan-3-ols) were quantified in TB teas (Table 2, Figure S1) by comparing their retention times and ultraviolet–visible light spectra with those of phenolic standards. The standard curves of phenolic compounds showed high correlations. Flavonols such as rutin and quercetin are the major phenolic compounds in TB [30,31]. In our study, rutin was the major compound in all of the teas, except in NRTB brewed at RTLT. Therefore, we identified rutin that reported high antiproliferative effects in human glioblastoma cells [32] might be responsible for the antiproliferative activities of TB teas. The rutin content in RTB brewed at RTLT was significantly higher than that in NRTB and RTB brewed at HTST (94.09 ± 17.41, 35.36 ± 1.10, and 40.26 ± 2.94 mg/g weight, respectively). Similarly, RTB brewed at RTLT showed the highest content of catechin (10.73 ± 2.47 mg/g weight), followed by RTB brewed at HTST, NRTB brewed at HTST, and NRTB brewed at RTLT (4.08 ± 0.82, 0.37 ± 0.08, and 0.14 ± 0.09 mg/g weight, respectively). Protocatechuic acid was the dominant phenolic compound in all TB teas except NRTB brewed at RTLT. These results are consistent with a previous report that protocatechuic acid is a major compound in TB [33]. The quercetin content of the NRTB brewed at HTST was significantly higher than that of the other TB teas. Quercetin suppresses the growth of breast, lung, and stomach cancer cell lines [34]. Thus, the difference in the antiproliferative activities of NRTB and RTB may be due to their different quercetin contents.

### 3.5. Correlation Analysis

**Figure 4:**
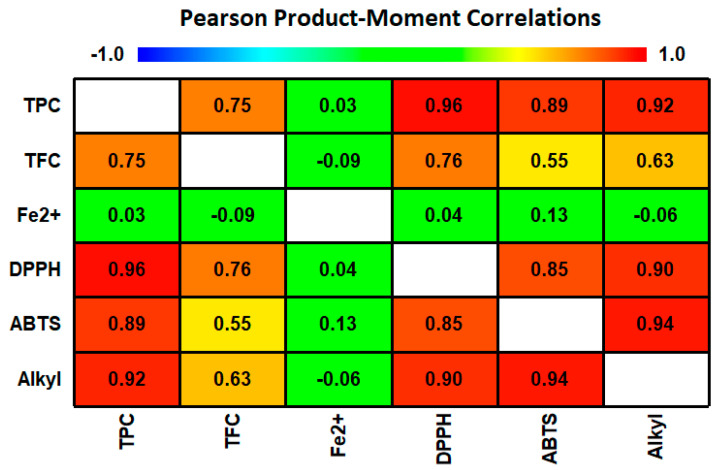
Correlations between the phenolic contents and antioxidant activities of TB teas. TPC, total polyphenol content; TFC, total flavonoid content.

A Pearson product-moment correlation analysis revealed strong positive relationships between TPC and the DPPH- (*r* = 0.96), ABTS- (*r* = 0.89), and alkyl-scavenging (*r* = 0.92) activities (Figure 4). In contrast, the TFC of the TB teas was positively correlated with their DPPH- (*r* = 0.76), ABTS- (*r* = 0.55), and alkyl-scavenging (*r* = 0.63) activities weakly. In contrast, the Fe^2+^-chelating activity of the TB teas was not correlated with their TPC, TFC, or free-radical-scavenging activities. In general, antioxidant activity is associated with phenolic compounds [35]. These results suggest that the antioxidant activity of the TB teas is due to their TPC rather than TFC.

## 4. Conclusions

We investigated the effects of the brew method and roasting on the phenolic content and antioxidant and antiproliferative activities of TB teas. The yields of RTLT extraction were higher than those of the HTST extraction. The TPC and TFC of RTB brewed at RTLT were higher than those of the other TB teas. The RTB brewed at RTLT showed DPPH-, ABTS-, and alkyl-scavenging activities, consistent with the TPC and TFC. In addition, the growth of human pancreatic cancer Panc-1 and breast cancer MDA-MB-231 cells was significantly inhibited in a dose-dependent manner by RTB brewed at RTLT. Rutin was the major compound in all extracts, except in NRTB brewed at RTLT. RTB brewed at RTLT showed the highest rutin, catechin, and protocatechuic acid contents. However, the quercetin content of the NRTB brewed at HTST was higher than that of the other extracts. RTB brewed at RTLT contained a higher rutin level and antioxidant activity than the other extracts did. Therefore, the RTLT method enables the production of functional beverages containing TB.

## Figures and Tables

**Table 1:** Extraction yield, and total polyphenol and flavonoid contents of TB teas.

Roasting Condition	Brew Method	Extraction Yield (%)	Total Polyphenol Content (mg GAE ^(1)^/g Dry Weight)	Total Flavonoid Content (mg RE ^(2)^/g Dry Weight)
NRTB ^(3)^	RTLT ^(4)^	2.12	3.58 ± 0.31 ^(5) a (6)^	1.86 ± 0.28 ^a^
	HTST	0.23	4.83 ± 0.40 ^b^	2.59 ± 0.51 ^a^
RTB	RTLT	4.81	11.07 ± 0.55 ^c^	6.03 ± 0.93 ^c^
	HTST	0.28	4.98 ± 0.44 ^b^	3.47 ± 0.84 ^b^

^(1)^ GAE, gallic acid equivalent, ^(^^2)^ RE, rutin equivalent. ^(3)^ NRTB, non-roasted Tartary buckwheat; RTB, roasted Tartary buckwheat. ^(4)^ RTLT, room temperature (25–30°C) for 24 h; HTST, high temperature (85–90°C) for 3 min. ^(5)^ Values are mean ± SD (*n* = 3). ^(6)^ Different letters represent significant differences among extracts, as determined by the least significant difference test at *p* < 0.05.

**Table 2:** Phenolic compound contents (mg/g dry weight) of TB teas.

(mg/g Dry Weight)	NRTB ^(1)^		RTB	
	RTLT ^(2)^	HTST	RTLT	HTST
Phenolic acids				
Gallic acid	0.04 ± 0.02 ^(^^3) a (4)^	0.06 ± 0.03 ^a^	1.00 ± 0.16 ^b^	0.44 ± 0.04 ^b^
Protocatechuic acid	0.20 ± 0.02 ^a^	4.00 ± 0.20 ^c^	5.11 ± 0.10 ^d^	1.49 ± 0.30 ^b^
4-Hydroxybenzoic acid	0.20 ± 0.01 ^a^	0.52 ± 0.03 ^c^	0.91 ± 0.04 ^d^	0.48 ± 0.03 ^b^
Flavonols				
Rutin	ND ^(5)^	35.36 ± 1.10 ^a^	94.09 ± 17.41 ^b^	40.26 ± 2.94 ^a^
Quercetin	1.04 ± 0.48 ^a^	154.87 ± 8.58 ^b^	1.18 ± 0.44 ^a^	1.09 ± 0.40 ^a^
Flavan-3-ols				
Catechin	0.14 ± 0.09 ^a^	0.37 ± 0.08 ^a^	10.73 ± 2.47 ^c^	4.08 ± 0.82 ^b^

^(1)^ NRTB, non-roasted Tartary buckwheat; RTB, roasted Tartary buckwheat. ^(2)^ RTLT, room temperature (25–30 °C) for 24 h; HTST, high temperature (85–90 °C) for 3 min. ^(3)^ Values are mean ± SD (*n* = 3). ^(4)^ Different letters represent significant differences among extracts, as determined by the least significant difference test at *p* < 0.05. ^(5)^ ND, not detected.

## Supplementary Materials

The following are available online at https://www.mdpi.com/2304-8158/9/9/1331/s1, Figure S1: HPLC chromatograms of TB teas.

## References

[1] Zhu F. (2016). Chemical composition and health effects of Tartary buckwheat. Food Chem..

[2] Wang L., Yang X., Qin P., Shan F., Ren G. (2013). Flavonoid composition, antibacterial and antioxidant properties of tartary buckwheat bran extract. Ind. Crop. Prod..

[3] Lee C.C., Shen S.R., Lai Y.J., Wu S.C. (2013). Rutin and quercetin, bioactive compounds from tartary buckwheat, prevent liver inflammatory injury. Food Funct..

[4] Sun T., Ho C.-T. (2005). Antioxidant activities of buckwheat extracts. Food Chem..

[5] Cai Y., Corke H., Li W., Wrigley C.W., Corke H., Walker C.E. (2004). Buckwheat. Encyclopedia of Grain Science.

[6] Birben E., Sahiner U.M., Sackesen C., Erzurum S., Kalayci O. (2012). Oxidative stress and antioxidant defense. World Allergy Organ. J..

[7] Reuter S., Gupta S.C., Chaturvedi M.M., Aggarwal B.B. (2010). Oxidative stress, inflammation, and cancer: How are they linked?. Free Radic. Biol. Med..

[8] Kulcharyk P.A., Heinecke J.W. (2001). Hypochlorous acid produced by the myeloperoxidase system of human phagocytes induces covalent cross-links between DNA and protein. Biochemistry.

[9] Thanan R., Oikawa S., Hiraku Y., Ohnishi S., Ma N., Pinlaor S., Shosuke K., Murata M. (2015). Oxidative stress and its significant roles in neurodegenerative diseases and cancer. Int. J. Mol. Sci..

[10] Santos C.N., Gomes A., Oudot C., Dias-Pedroso D., Rodriguez-Mateos A., Vieira H.L., Brenner C. (2018). Pure polyphenols applications for cardiac health and disease. Curr. Pharm. Des..

[11] Fuller M., Rao N.Z. (2017). The Effect of Time, Roasting Temperature, and Grind Size on Caffeine and Chlorogenic Acid Concentrations in Cold Brew Coffee. Sci. Rep..

[12] Xu Y.-Q., Zou C., Gao Y., Chen J.-X., Wang F., Chen G.-S., Yin J.-F. (2017). Effect of the type of brewing water on the chemical composition, sensory quality and antioxidant capacity of Chinese teas. Food Chem..

[13] Liu Y., Luo L., Liao C., Chen L., Wang J., Zeng L. (2018). Effects of brewing conditions on the phytochemical composition, sensory qualities and antioxidant activity of green tea infusion: A study using response surface methodology. Food Chem..

[14] Pérez-Burillo S., Giménez R., Rufián-Henares J.A., Pastoriza S. (2018). Effect of brewing time and temperature on antioxidant capacity and phenols of white tea: Relationship with sensory properties. Food Chem..

[15] Lin S.-D., Liu E.-H., Mau J.-L. (2008). Effect of different brewing methods on antioxidant properties of steaming green tea. LWT Food Sci. Technol..

[16] Rao N.Z., Fuller M. (2018). Acidity and Antioxidant Activity of Cold Brew Coffee. Sci. Rep..

[17] Saklar S., Ertas E., Ozdemir I.S., Karadeniz B. (2015). Effects of different brewing conditions on catechin content and sensory acceptance in Turkish green tea infusions. J. Food Sci. Technol..

[18] Cheung L.M., Cheung P.C., Ooi V.E. (2003). Antioxidant activity and total phenolics of edible mushroom extracts. Food Chem..

[19] Zhishen J., Mengcheng T., Jianming W. (1999). The determination of flavonoid contents in mulberry and their scavenging effects on superoxide radicals. Food Chem..

[20] Blois M.S. (1958). Antioxidant Determinations by the Use of a Stable Free Radical. Nature.

[21] Brand-Williams W., Cuvelier M.E., Berset C. (1995). Use of a free radical method to evaluate antioxidant activity. LWT Food Sci. Technol..

[22] Re R., Pellegrini N., Proteggente A., Pannala A., Yang M., Rice-Evans C. (1999). Antioxidant activity applying an improved ABTS radical cation decolorization assay. Free Radic. Biol. Med..

[23] Hiramoto K., Johkoh H., Sako K., Kikugawa K. (1993). DNA breaking activity of the carbon-centered radical generated from 2,2′-azobis(2-amidinopropane) hydrochloride (AAPH). Free Radic. Res. Commun..

[24] Carmichael J., DeGraff W.G., Gazdar A.F., Minna J.D., Mitchell J.B. (1987). Evaluation of a tetrazolium-based semiautomated colorimetric assay: Assessment of chemosensitivity testing. Cancer Res..

[25] Elsorady M., Ali S. (2018). Antioxidant activity of roasted and unroasted peanut skin extracts. Int. Food Res. J..

[26] Nam J.-S., Jang H.-L., Rhee Y.H. (2017). Antioxidant Activities and Phenolic Compounds of Several Tissues of Pawpaw (*Asimina triloba* [L.] Dunal) Grown in Korea. J. Food Sci..

[27] Chinnapun D. (2018). Antioxidant activity and DNA protection against oxidative damage of bambara groundnut seeds (*Vigna subterranea* (L.) Verdc.) as affected by processing methods. Int. J. Food Prop..

[28] Guo R., Chang X., Guo X., Brennan C.S., Li T., Fu X., Liu R.H. (2017). Phenolic compounds, antioxidant activity, antiproliferative activity and bioaccessibility of Sea buckthorn (*Hippophae rhamnoides* L.) berries as affected by in vitro digestion. Food Funct..

[29] Li F., Zhang X., Li Y., Lu K., Yin R., Ming J. (2017). Phenolics extracted from tartary (*Fagopyrum tartaricum* L. Gaerth) buckwheat bran exhibit antioxidant activity, and an antiproliferative effect on human breast cancer MDA-MB-231 cells through the p38/MAP kinase pathway. Food Funct..

[30] Qin P., Wu L., Yao Y., Ren G. (2013). Changes in phytochemical compositions, antioxidant and α-glucosidase inhibitory activities during the processing of tartary buckwheat tea. Food Res. Int..

[31] Fabjan N., Rode J., Kosir I.J., Wang Z., Zhang Z., Kreft I. (2003). Tartary Buckwheat (Fagopyrum tataricum Gaertn.) as a Source of Dietary Rutin and Quercitrin. J. Agric. Food Chem..

[32] Santos B.L., Silva A.R., Pitanga B.P.S., Sousa C.S., Grangeiro M.S., Fragomeni B.O., Coelho P.L.C., Oliveira M.N., Menezes-Filho N.J., Costa M.F.D. (2011). Antiproliferaive, proapoptotic and morphogenic effects of the flavonoid rutin on human glioblastoma cells. Food Chem..

[33] Guo X.-D., Ma Y.-J., Parry J., Gao J.-M., Yu L.-L., Wang M. (2011). Phenolics content and antioxidant activity of tartary buckwheat from different locations. Molecules.

[34] Hashemzaei M., Delarami Far A., Yari A., Heravi R.E., Tabrizian K., Taghdisi S.M., Sadegh S.E., Tsarouhas K., Kouretas D., Tzanakakis G. (2017). Anticancer and apoptosis-inducing effects of quercetin in vitro and in vivo. Oncol. Rep..

[35] Kim Y.H., Cho M.L., Kim D.B., Shin G.H., Lee J.H., Lee J.S., Park S.-O., Lee S.-J., Shin H.M., Lee O.H. (2015). The antioxidant activity and their major antioxidant compounds from Acanthopanax senticosus and A. koreanum. Molecules.

