# Supplementary material for: Effect of Roasting and Brewing on the Antioxidant and Antiproliferative Activities of Tartary Buckwheat
Source: Foods. 2020 Sep 21;9(9):1331. doi: 10.3390/foods9091331 (PMC7555746; doi:10.3390/foods9091331)
Supplement: Supplementary file 1 [file foods-09-01331-s001.pdf]

Effect of Roasting and Brewing on the Antioxidant and Antiproliferative Activities of Tartary Buckwheat

Ji-yeon Ryu [1,†], Yoonseong Choi [2,†], Kun-Hwa Hong [3], Yong Suk Chung [4] and Somi Kim Cho [1,5*]

[1] School of Biomaterials Sciences and Technology, College of Applied Life Sciences, SARI, Jeju National University, Jeju 63243, Korea; rjo211@naver.com

[2] Lucy Cavendish College, University of Cambridge, Cambridge CB3 0BU, UK; yc472@cam.ac.uk

[3] With O co., Jeju 63309, Korea; witho62@naver.com

[4] Department of Plant Resources and Environment, Jeju National University, Jeju 63243, Korea; yschung@jejunu.ac.kr

[5] Interdisciplinary Graduate Program in Advanced Convergence Technology and Science, Jeju National University, Jeju 63243, Korea

* Correspondence: phd.kim.somi@gmail.com; Tel.: +82-64-754-3348

† These authors contributed equally to this work

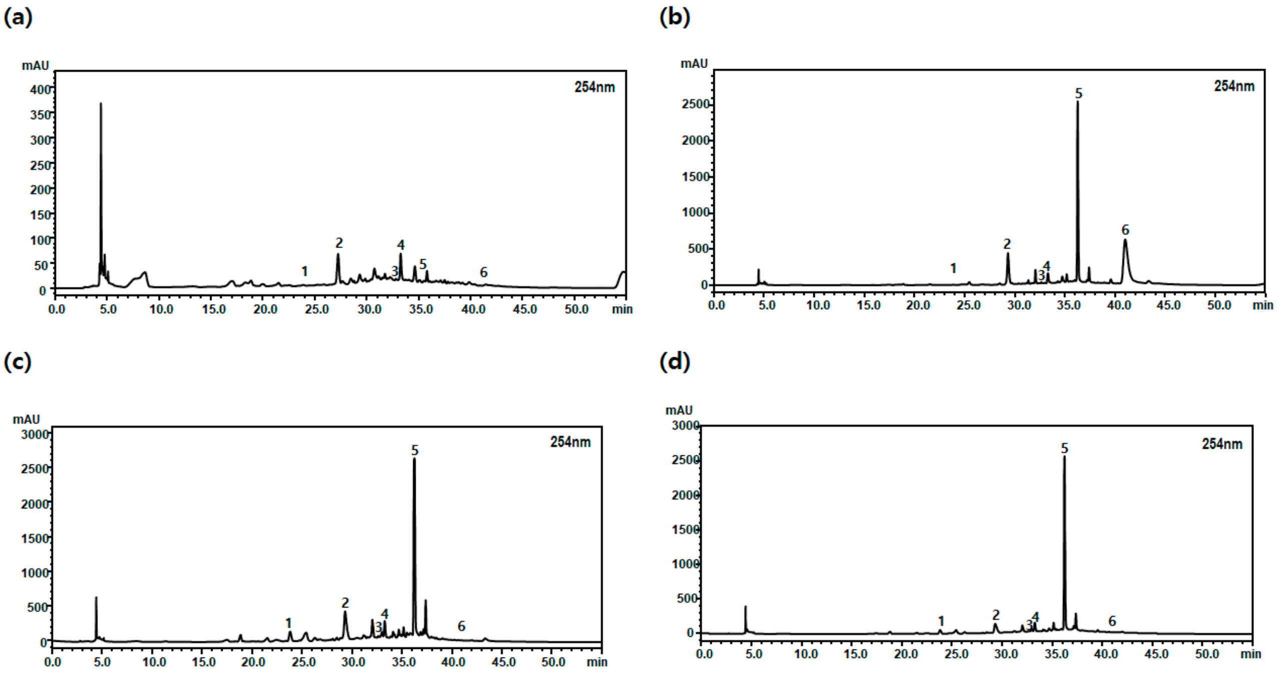

Supplementary Fig. S1. HPLC chromatograms of TB teas. (**a**) NRTB-RTLT, (**b**) NRTB-HTST, (**c**) RTB-RTLT, and (**d**) RTB-HTST. NRTB, non-roasted Tartary buckwheat; RTB, roasted Tartary buckwheat. RTLT, room temperature (25–30 °C) for 24 h; HTST, high temperature (85–90 °C) for 3 min.; 1, gallic acid; 2, protocatechuic acid; 3, catechin; 4, 4-hydroxybenzoic acid; 5, rutin; 6, quercetin.
